# Correlates of Parental Misperception of Their Child’s Weight Status: The ‘Be Active, Eat Right’ Study

**DOI:** 10.1371/journal.pone.0088931

**Published:** 2014-02-14

**Authors:** Teun Remmers, Amy van Grieken, Carry M. Renders, Remy A. Hirasing, Suzanne M. L. Broeren, Hein Raat

**Affiliations:** 1 Department of Public Health, Erasmus MC University Medical Center Rotterdam, Rotterdam, the Netherlands; 2 Department of Public and Occupational Health, EMGO Institute of Health and Care Research, VU University Medical Center Amsterdam, Amsterdam, the Netherlands; 3 Institute of Health Sciences, Faculty of Earth and Life Sciences, VU University Amsterdam, Amsterdam, the Netherlands; 4 Department of Epidemiology, CAPHRI School for Public Health and Primary Care, Maastricht University Medical Center, Maastricht, the Netherlands; Scientific Directorate, Bambino Hospital, Italy

## Abstract

**Objective:**

This study reported on correlates of parental perception of their child’s weight status. Associations between parental misperception (i.e., underestimation of the child’s weight) and parental intention to improve their child’s overweight-related health behaviors and their child meeting guidelines regarding these behaviors were also investigated.

**Methods:**

Baseline data from the population-based ‘Be active, eat right study’ were used. The population for analysis consisted of 630 overweight and 153 obese five year-old children and their parents. Questionnaires were used to measure parental perception of the child’s weight status, correlates of misperception (i.e., child age, child gender, child BMI, parental age, parental gender, parental country of birth, parental educational level and parental weight status), overweight-related health behaviors (i.e., child playing outside, having breakfast, drinking sweet beverages, and watching TV), and parental intention to improve these behaviors. Height and weight were measured using standardized protocols. Multivariable logistic regression analyses were performed.

**Results:**

In total, 44.40% of the parents misperceived their child’s weight status. Parental misperception was associated with lower child BMI, the parent being the father, a foreign parental country of birth, and a lower parental education level (*p*<0.05). Parental misperception was not associated with parental intention to improve child overweight-related health behavior, nor with child meeting the guidelines of these behaviors.

**Discussion:**

This study showed that almost half of the parents with an overweight or obese child misperceived their child’s weight status. A correct parental perception may be a small stepping-stone in improving the health of overweight and obese children.

## Introduction

Parents have great influence on children’s overweight-related health behaviors, such as physical activity, having breakfast, drinking sweet beverages, and watching TV [1], [2]. By promoting or restricting these behaviors, parents have a crucial role in the prevention and treatment of overweight in children [3].

An important first step for the successful involvement of parents in prevention and treatment programs for overweight and obese children is a realistic parental perception of their child’s weight status [4]. An underestimation of their child’s weight status (and thus an unrealistic parental perception) is defined as ‘parental misperception’. A recent meta-analysis on parental perception of child’s weight status [5], showed that approximately 62.4% of the parents of overweight or obese children misperceived their child as having normal weight. This meta-analysis also showed that parental misperception was higher in 2–6 year-old compared to 7–18 year-old children. This may be of interest to interventions focusing on parental involvement in the treatment of overweight and obese children, as this misperception is a first threat to the success of these interventions. Early interventions in overweight children will potentially yield in long-term benefits as early childhood significantly predicts long-term overweight risks [6], [7]. Also, parental influence is considered to be relatively high for young children’s behavior [8], [9], and overweight-related health malignancies such as decreased insulin sensitivity and elevated blood pressure are already seen in young children [10].

Parental misperception may influence parental actions to improve children’s overweight-related health behaviors. Therefore, parental misperception is especially relevant in children that are already overweight or obese. Studies that investigate correlates of parental misperception are warranted for defining sub-populations at higher risk of parental misperception. However, little is known about associations between parental misperception and parents’ intentions to improve their child’s overweight-related health behaviors, and their child’s actual engagement in overweight-related health behaviors. One study [11] reported that approximately one third of the parents of an overweight or obese child had taken no action, half of these parents limited snack consumption, and only 3.5% and 5% considered limiting sweet drinks consumption and increasing physical activity, respectively. Furthermore, one study reported that parental misperception was not associated with parent perceived child nutrition [12], another study reported that a parental perception was associated with maternal plans to control their child’s weight [13]. If parental misperception would indeed be associated with these parental plans and therefore indirectly related to less favorable child engagement in overweight-related health behaviors, parental misperception may be targeted in the treatment of childhood overweight and obesity.

This study will investigate whether parents that misperceive their child’s weight status differ in the management of their child’s overweight, compared to parents with a realistic perception. More specifically, we will investigate correlates of parental misperception, associations between parental misperception and parental intention to improve child’s overweight-related health behaviors, and associations between parental misperception and the probability of children meeting guidelines regarding overweight-related health behaviors. We hypothesize that parental misperception of child’s weight status is associated with a relatively low probability parental intention to improve their child’s engagement in overweight-related health behaviors, compared to those with a correct parental perception. Furthermore, we hypothesize that parental misperception is related to a relatively low probability of meeting the guidelines, compared to parents with a correct parental perception.

## Methods

### Study Design and Population

The present cross-sectional study was embedded in the ‘Be active, eat right’ study, a cluster randomized controlled trial evaluating an overweight prevention program among five to seven year-old children (Current Controlled Trials ISRCTN04965410). The study is described in detail elsewhere [14]. The Medical Ethics Committee of the Erasmus University Medical Center approved the study protocol (ref nr. MEC-2007-163). In total, 13,638 parents of five year-old children were invited to participate in the ‘Be active, eat right’ study. Parents provided written informed consent for participation in the two-year study (*N* = 8784; participation percentage 64%).

**Figure 1:**
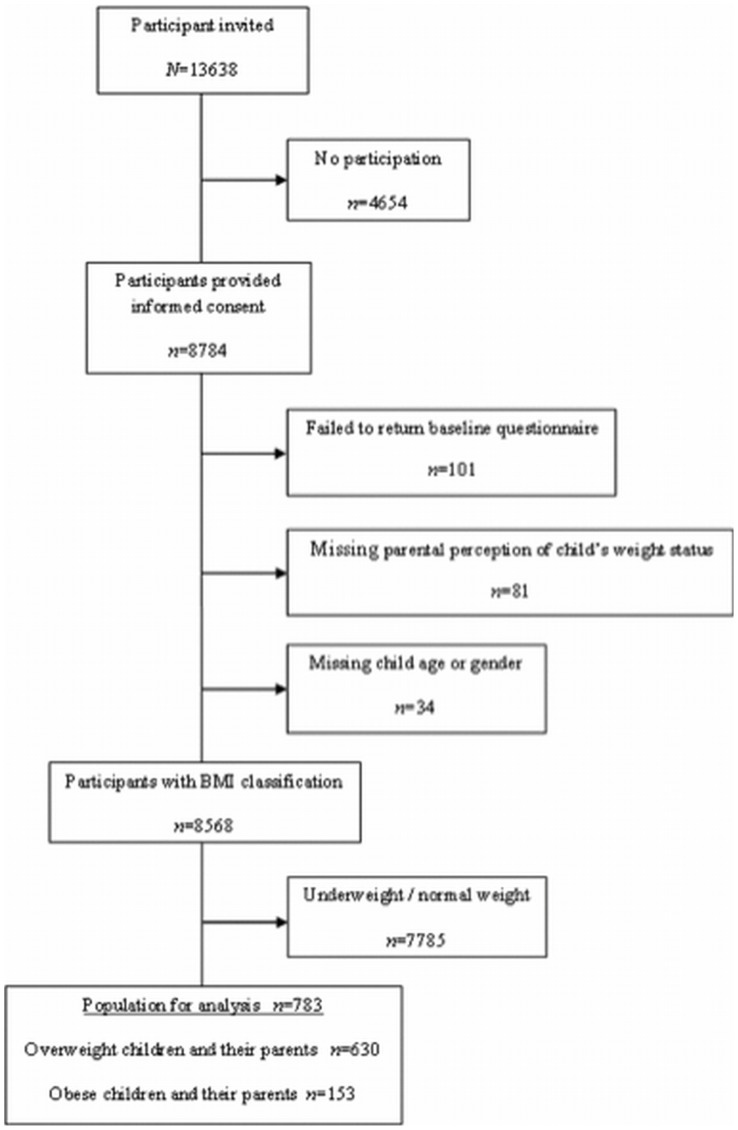
Flowchart.

For this study, the intervention and control group were combined, and only baseline measurements (*N* = 8784; see Figure 1) were used. Both conditions could be combined because the intervention in study commenced after the baseline measurements. The baseline questionnaires assessing parental perception of child’s weight status were filled in by the parents (both fathers and mothers, hereafter called parents) a few weeks prior to the height and weight measurements, so that the parents were unaware of their child’s measured weight status at the time of the assessment of their perception of their child’s weight. We excluded participants with missing values on the variables assessing the perception of child’s weight status, child’s age, gender, and Body Mass Index (BMI). After selecting the subgroup of parents with overweight or obese children, the population for analysis consisted of 783 children and their parents (see Figure 1 for flowchart).

In total, nine youth health care (YHC) centers were included in the study, which consisted of 44 YHC teams. The YHC teams were the unit of randomization. In the present study’s population for analyses (n = 783 participants of the control condition) 43 YHC teams consisted of at least one participant. In contrast to studies that examine an intervention effect of a cluster RCT, sampling of participants in the present study was independent of the clusters, and the analyses of the present study were not based on the cluster division. To evaluate whether potential clustering sampling influenced our results, we additionally applied cluster-corrected mixed models (see supplemental material, Table S2). The survey questionnaire consisted of validated components (e.g., SQUASH [15]), and was based on existing questionnaires assessing health behavior in children [16].

### Measurements

#### Parental perception of child’s weight status

Parental perception of their child’s weight status was assessed by the question: “If you compare your child with other children having equal height and gender, do you think your child’s weight is lighter or heavier?” The five response categories were: *much lighter*, *lighter*, *equal, heavier*, and *much heavier*. Because the sample for analyses only contained overweight and obese children, all parent’s perceptions that their child was (much) lighter or of equal weight were categorized as misperception, and the responses indicating that their child was heavier or much heavier were categorized as a realistic perception.

#### Correlates of parental misperception

Based on results from previous studies [4], [17]–[20], several potential correlates of parental misperception were included in this study (i.e., child age, child gender, child BMI, parental age, parental gender, parental country of birth, parental educational level and parental weight status). Child height and weight were measured by trained youth health care professionals, utilizing standardized methods according to the Dutch growth study [21]. Child BMI (weight in kilograms divided by height in meters squared) was calculated and categorized into overweight and obesity, using international thresholds standardized for age and gender [22].

The parent reported his or her own current height, weight, gender, and further socio-demographics in the baseline questionnaire. Parent BMI was computed, and parents were categorized as normal weight or underweight versus parents overweight (including obesity). Furthermore, parent’s country of birth was categorized as ‘Dutch’ or ‘non-Dutch’ (i.e., foreign country of birth). Parent educational level was assessed by the highest completed level of education; categorized as low (no education, primary school, or ≤3 years of general secondary school), mid-low (>3 years of general secondary school), mid-high (higher vocational training, undergraduate programs, or bachelor’s degree), and high (higher academic education) [23].

#### Parental intention to improve child’s overweight-related health behaviors

Parental intention to improve child’s overweight-related health behaviors was conceptualized as the intention to increase their child’s engagement in playing outside and having daily breakfast, and decrease their child’s engagement in drinking sweet beverages and watching TV in the upcoming half-year. This was assessed with the statement “In the upcoming half-year, it is my intention to let my child … (e.g., play outside more, etc.)”. The five response categories were *totally agree*, *agree, do not agree/do not disagree*, *disagree*, and *totally disagree*. Parental intention was dichotomized in parents that agreed with the intention (*totally agree and agree*) versus parents that did not agree with intention (*do not agree/do not disagree*, *disagree*, and *totally disagree*) for all four overweight-related health behaviors separately.

#### Child’s actual engagement in overweight-related health behaviors

First, parents reported the average time per day (in hours and minutes) that their child played outside, separately for week and weekend days. A daily average was computed and dichotomized according to the child meeting the Dutch recommendation of physical activity of one hour or more per day versus not meeting this guideline [2]. Second, the number of days that the child had breakfast in an average/normal week was assessed. Response categories were one to seven days per week. Categories were dichotomized in either having daily breakfast (i.e., seven days per week) or not having breakfast daily (i.e., all other response categories). Third, child’s intake of sweet drinks (i.e., glasses of lemonade, [carbonated] soft drinks, juices, chocolate drinks) on an average day was assessed. Response categories consisted of *none/less than 1, 1–2, 3–4, 5–6, 7–8*, and *9 or more glasses* per day. Categories were dichotomized into drinking less or equal than two drinks per day versus drinking more than two drinks, based on earlier research using the distribution in the data [24]. Finally, parents reported the average time per day (in hours and minutes) that their child watched TV, separately for week and weekend days. Subsequently, a daily average of watching TV was computed, and dichotomized in either less or equal than two hours per day or watching more than two hours per day, according to Dutch recommendations and the guidelines of the American Academy of Pediatrics [25], [26].

### Data Analyses

First, descriptive statistics for all variables of interest were computed for the total population, and separately for the parents that misperceived their child’s weight status versus the parents that correctly perceived their child’s weight status (Table 1). Univariate differences between these subgroups were tested for statistical significance with chi-square in the case of categorical variables and independent samples *t*-tests in the case of continuous variables.

**Table 1:** Child and parental characteristics (*n* = 783).

	Total population (*n* = 783)	Misperception (*n* = 348)	Correct perception (*n* = 435)
**Child characteristics**			
Age in years (mean, SD)	5.76 (0.43)	5.74 (0.43)	5.78 (0.43)
Gender (% girls)	60.70	60.10	61.10
BMI in kg/m^2^ (mean, SD)	18.79 (1.41)	18.29 (0.96)*	19.19 (1.57)*
Weight status (% Obese)	19.54	8.30*	28.50*
**Parental characteristics**			
Age in years (mean, SD) (missing *n* = 5)	36.45 (5.26)	36.38 (5.98)	36.52 (4.63)
Gender (% female) (missing *n* = 8)	88.50	83.50*	92.40*
Country of birth (% Dutch)	84.40	76.00*	91.00*
Educational level (missing *n* = 16)		*	*
% Low	5.60	7.90	3.70
% Mid-low	27.20	31.80	23.50
% Mid-High	44.40	42.60	45.30
% High	22.90	17.80	27.00
Weight status (% overweight/obese) (missing *n* = 30)	48.20	52.10	45.20

Note: if the number of missing values is not reported, missing *n* = 0.

*Significant difference between subgroup of misperception versus correct perception at *p*<0.05 using *t*-test for continuous variables and chi-square for categorical variable.

Second, a multivariable logistic regression analysis was performed to evaluate correlates of parental misperception of child’s weight status (Table 2). Parental misperception (versus correct parental perception) was the dependent variable. Child age, child gender, child BMI, parental age, parental gender, parental country of birth, parental educational level, and parental weight status were the independent variables. We presented 1) the crude association of each individual independent variable, 2) the association of each independent variable after selecting influential confounders from all variables in Table 1 according change in estimate strategy [27], and 3) the independent association when adjusting for all correlates independent of their statistical significance.

**Table 2:** Multivariable logistic regression analysis, presenting Odds Ratio’s (OR) for correlates of parental misperception about child’s weight status.

	Crude OR (95% C.I.)	Adjusted OR (95% C.I.)‡	Adjusted OR (95% C.I.)^†^
**Child characteristics**			
Age (years)	0.79 (0.57 to 1.10)	=	0.87 (0.60 to 1.28)
Girls (boy = reference)	0.96 (0.72 to 1.28)	0.79 (0.57 to 1.07)	0.91 (0.65 to 1.28)
BMI (kg/m^2^)	0.53 (0.46 to 0.62)*	=	0.47 (0.39 to 0.56)*
**Parental characteristics**			
Age (years)	1.00 (0.97 to 1.02)	=	0.99 (0.96 to 1.03)
Female (male = reference)	0.42 (0.27 to 0.66)*	0.40 (0.24 to 0.66)*	0.42 (0.24 to 0.73)*
Foreign country of birth (Dutch = reference)	3.20 (2.12 to 4.82)*	4.98 (3.08 to 8.04)*	3.93 (2.29 to 6.74)*
Educational level (high = reference)			
Low educational level	3.21 (1.61 to 6.41)*	6.03 (2.75 to 13.26)*	3.00(1.21 to 7.43)*
Mid-low educational level	2.05 (1.36 to 3.10)*	2.65 (1.70 to 4.12)*	2.53 (1.58 to 4.06)*
Mid-high educational level	1.41 (0.97 to 2.05)	1.63 (1.09 to 2.42)*	1.50 (0.98 to 2.30)*
Overweight/obese weight status(normal/underweight = reference)	1.32 (0.99 to 1.76)	1.69 (1.24 to 2.31)*	1.38 (1.00 to 1.93)

‡ adjusted for selected variables according to ‘change in estimate’ strategy, ^†^all correlates are corrected for each other,  =  none of the correlates led to a change in estimate of ≧10% and therefore the adjusted estimate is equal to the crude estimate, *significant difference between subgroup of misperception versus correct perception at *p*<0.05.

Third, multivariable logistic regression analyses were also performed to evaluate associations between parental misperception and parental intentions to improve their child’s engagement in overweight-related health behaviors. In addition, in these analyses, associations between parental misperception and the child meeting the guidelines regarding overweight-related health behaviors were studied as well. The same analytical strategy was used as for the results of Table 2 (see paragraph above). For the results of this procedure, please see Table S1. Potential effect modification on the association between parental intentions and parental misperception was explored by including interaction terms between the covariate and all statistically significant correlates in Table 2. None of the interactions were statistically significant and therefore none of the interaction terms were further explored or entered in the final analyses. Statistical significance was assumed at *p*<0.05 in all analyses. All analyses were performed with SPSS [28].

## Results

### Child and Parental Characteristics

Table 1 shows the characteristics of the study population. Of the children, 60.7% were girls, and the average age was 5.8 years (*SD* = 0.4). All children were overweight or obese. Of the parents, 88.5% was female; their average age was 36.5 (*SD* = 5.2) years. Forty eight percent of the parents were overweight themselves. Finally, 44.4% of the parents misperceived their child’s weight status; this was 50.6% for parents of overweight children, and 19.0% for parents of obese children (data not shown in Table 1).

To evaluate potential clustering sampling, we applied cluster-adjusted mixed model analyses, with cluster (i.e., YHC team) entered as a random factor (see Table S2). As these results did not differ, we decided to present Table 3 in this manuscript.

**Table 3:** Logistic regression analyses, presenting the association between parental misperception and 1) parental intention to improve child engagement in overweight-related health behaviors, and 2) child meeting guidelines regarding these behaviors.

Parental misperception and parental intention(agreed vs. not agreed with intention)		Playing outside	Having daily breakfast	Drinking sweet beverages	Watching TV
	Crude OR (95% CI)	0.85 (0.58 to 1.23)	1.13 (0.77 to 1.64)	0.93 (0.64 to 1.36)	1.07 (0.73 to 1.55)
	Adjusted OR (95% CI)‡	0.83 (0.55 to 1.25)	1.01 (0.67 to 1.33)	0.90 (0.60 to 1.36)	0.94 (0.62 to 1.43)
	Adjusted OR (95% CI)^†^	0.81 (0.52 to 1.24)	0.94 (0.61 to 1.45)	0.82 (0.54 to 1.27)	0.92 (0.60 to 1.43)
**Parental misperception and child meeting guidelines (meeting guideline vs. not meeting guideline)**		**Playing outside** **≥1 hour per day**	**Having daily** **breakfast**	**Drinking ≤2 sweet beverages per day**	**Watching ≤2 hours of TV per day**
	Crude OR (95% CI)	1.18 (0.63 to 2.22)	0.53 (0.33 to 0.83)*	0.98 (0.72 to 1.34)	0.69 (0.49 to 0.96)*
	Adjusted OR (95% CI)‡	1.43 (0.74 to 2.75)	0.57 (0.34 to 0.94)*	0.98 (0.72 to 1.34)	0.67 (0.46 to 0.97)*
	Adjusted OR (95% CI)^†^	1.29 (0.64 to 2.60)	0.60 (0.36 to 1.01)	0.91 (0.64 to 1.28)	0.85 (0.57 to 1.27)

‡ adjusted for selected variables according to ‘change in estimate’ strategy, ^†^adjusted for child age, gender, BMI and parental age, gender, country of birth, educational level, and weight status, *significant difference at *p*<0.05.

### Correlates of Parental Misperception

Within all presented analytical strategies in Table 2, child BMI, parental gender, parental country of birth, and parental educational level were related to parental misperception. More specifically, an increment of one BMI point was associated with lower odds of parental misperception (OR = 0.47, 95% CI = 0.39–0.56). Mothers had lower odds to misperceive their child’s weight status than fathers (OR = 0.42, 95% CI = 0.24–0.73), and parents with a non-Dutch country of birth had higher odds of misperception compared to Dutch parents. Furthermore, lower educational level was associated with higher odds of parental misperception compared to a highest academic education (OR = 3.00, 2.53, 1.50 for low, mid-low and mid-high education levels, respectively). A trend was observed for parents who were overweight or obese themselves, with this group showing a somewhat higher probability of misperceiving their child’s weight status compared to parents that were not overweight or obese (95% CI of OR = 1.00–1.93). This association was significant when the number of confounders were selected based on their relative influence.

### Parental Misperception and Parental Intention

In total, 42.5% of the parents expressed their intention to increase their child’s engagement in playing outside, whereas 34.0% expressed their intention to have daily breakfast. Regarding decreasing the consumption of sweet beverages and watching TV, respectively 36.6% and 51.0% of the parents expressed their intention to improve on these behaviors (data not shown). The content of the analyses in which confounders were selected according to their relative influence on the relationship (see ‡ in Table 3), can be found in Table S1. Parental misperception was not associated with parent’s intention to improve engagement in any of the overweight-related health behaviors (see Table 3).

### Parental Misperception and Child Meeting Guidelines

In total, 93.80% of the children met the daily guideline regarding playing outside. For having breakfast, this percentage was 88.40%, for watching TV 72.60%, whereas for drinking sweet beverages only 32.70% of the children met the guideline (data not shown). Again, the content of the analyses in which confounders were selected according to their relative influence on the relationship (see ‡ in Table 3), can be found in Table S1. Except for guidelines regarding breakfast and watching TV in first adjusted model (‡), parental misperception was not associated with meeting the guidelines in any of the overweight-related health behaviors (see Table 3).

## Discussion

Almost half of the parents with an overweight (including obesity) child in this study misperceived their child’s weight status. Higher probabilities of parental misperception were found for children with a lower child BMI, when the father reported on the child’s weight status, when the parent was from a foreign country of birth or had a lower educational level. Parental misperception was not associated with the intention to improve child engagement in overweight-related health behaviors or meeting guidelines regarding overweight-related health behaviors.

The percentage of parental misperception found in this study was relatively low compared to the pooled percentage estimate from a recent meta-analysis (range 60–65%) [5]. This could be due to differences in assessment of parental misperception. We specifically asked parents to compare their child with other children of the same height and gender. Various other studies used of the phrase “overweight” in their question to assess parental misperception [4], [29]–[31]. The use of the phrase “overweight” may influence estimations; parents perceive difficulties in understanding the term overweight [32] and are hesitant to label their child as overweight [33]. On the other hand, our findings may be influenced by the reference parents have when comparing their child with other children of the same height and gender [34], [35]. Taken together, our study suggests that without specifically phrasing “overweight” in the question to the parents, the percentage of parental misperception was lower compared to other studies. However, despite this improvement in correct perception, still almost half of the parents with an overweight or obese child misperceived their child’s weight status.

In line with previous studies [19], [35], [36], we found a higher percentage of parental misperception in overweight children, compared to obese children. This is plausible, as in obese children, overweight is more visually apparent. Furthermore, in the present study, no difference in parental misperception was found between boys and girls. Previous studies investigating gender differences have reported conflicting results. For example, some studies found no difference in parental misperception between boys and girls [17], [37], [38], while other studies found that parents were more likely to misperceive boys’ weight status compared to girls’ [39], [40]. Therefore, future studies that investigate this are warranted.

In line with a previous study [39], we also found that fathers were more likely to misperceive their child’s weight status than mothers. However, the reason for this difference is still unclear. Future studies are recommended to further investigate discrepancies in results regarding misperception of child’s weight status between mothers and fathers.

Moreover, our study showed that both lower educational level and a foreign country of birth were independently associated with parental misperception. Findings regarding educational level were in line with a study of Baughcum et al (2000), which reported that lower educational level was independently associated with relatively high parental misperception. With regard to country of birth of the parent, previous studies have shown that specific ethnic minority groups (i.e., Latino/Hispanic [11], [41], [42] and African American parents [20]), often fail to identify their child’s overweight as a problem. It could be that both findings regarding educational level and country of birth are due to a cultural difference in overweight risk perception [43] and/or parental reference regarding child overweight between Dutch and non-Dutch parents [34], [35]. This latter suggestion is supported by the fact that non-Dutch parents have often been shown to have a lower education level [44] and overweight and obesity are more prevalent in non-Dutch groups [45]–[47]. Therefore, the references group the non-Dutch parents use may be different (i.e., heavier) than the reference group of the Dutch parents.

Furthermore, approximately one third to half of the parents expressed their intention to improve their child’s overweight-related health behaviors, irrespective of their perception. Contrary to our hypothesis, no association was found between parental misperception and the parental intention to improve child’s overweight-related health behaviors. Perhaps a correct parental perception of a child’s overweight or obesity is not a necessary prerequisite for parental intention to improve overweight-related health behaviors, as these behaviors may be more universal to all parents (i.e. most parents want their child to be healthy). Another explanation may be that parents have unintentionally been triggered in their intention to improve their child’s overweight-related behaviors, by solely filling out questionnaires regarding their child’s health and their engagement in healthy behaviors. Again, our results may have been attenuated by this phenomenon. Therefore, it is still unclear whether interventions should focus on parental misperception as part of a primary intervention strategy.

In addition to intention towards improving child’s overweight-related health behaviors, child’ actual overweight-related health behaviors were also not associated with parental misperception. The latter finding was supported by the studies of Myers et al (2000) and Genovesi et al (2005). According to the parental reports, the percentage of children that meet the guidelines of overweight-related health behaviors were higher than expected, as these children were all overweight or obese. Parents of overweight children in general (irrespective of their perception of their child’s weight status) may have misperceived their child’s engagement in overweight-related health behaviors as they are e.g. not always aware of their children’s behavior during and after school, and this misclassification may have attenuated a potential association between parental misperception and children meeting the guidelines of overweight-related health behaviors. Future studies investigating this relationship are urged to use objective measurements of these behaviors (such as accelerometers) or by using observations. As we used a cross-sectional design, we were unable to investigate implications of parental misperception on overweight-related health behaviors over time.

### Strengths and Limitations

Strength of the present study is its relatively large sample of overweight and obese children. Another strength is the multivariable regressions, allowing for the interpretation of associations independent of confounding between predictors and covariates. However, there are also limitations. Due to the cross-sectional design, directionality of associations concerning parental misperception could not be determined.

The percentage of overweight and obesity in our population for analyses is somewhat smaller than the Dutch reference population [48], [49], which may be indicative for selection bias. This limits the generalization of our study’s results, and may be caused by the possibility that parents of overweight or obese children were more likely to refuse participation at initiation of the study. In addition, above we showed that parents from overweight children provided more missing values on our primary variables of interest, which may have overestimated the percentage of parents with parental misperception, as BMI was negatively associated with parental misperception. Whether this selective sampling also affected the associations at hand is questionable, because this also depends on the characteristics of our subsample regarding our outcome variables (i.e., overweight-related health behaviors). In addition, no studies to date have evaluated the reliability and validity of parental misperception assessment. According to the results of the present study however, one can suggest that specifically labeling children as “overweight” is associated with a smaller percentage of parental misperception, which may help researchers to validate relevant questions regarding parental misperception in the future. Some selective dropout of participants was found concerning the measurements of parental intention and overweight-related health behaviors; parents with missing values on parental misperception had higher parental intention towards playing outside, drinking sweet beverages, and watching TV. Therefore, the results should be interpreted and generalized with caution.

Nonetheless, we provided a cluster corrected analyses of Table 3 in the supplemental material, which supported our view that results of this study were not affected by clustering sampling (Table S2). Finally, the overweight-related health behaviors in this study were only assessed by parental report. As previous studies have shown that parent-report of child behavior can be biased [50], [51], future studies also need to include objective measurements of health behaviors (e.g., accelerometers or video observations).

## Conclusion

This study showed that less than half of the parents with an overweight or obese child misperceived their child’s weight status. Parental misperception was positively associated with lower child BMI, the father reporting on the child’s weight status, the parent not being born in the Netherlands, and the parent being lower educated. According to the transtheoretical model, correct parental perception of overweight and obesity is considered as a first step in the treatment of overweight and obese children. However, in our study we could not confirm an association between parental misperception and intention to change child health behavior. In line herewith we observed no association between parental misperception and the child’s actual engagement in health-related behaviors.

Research should also take into account the potential influences of a foreign background and educational level. A correct parental perception may be a small stepping-stone in improving the health of overweight and obese children. In the meantime, health care practitioners should focus on informing and motivating parents on how to promote healthy behaviors.

## Supporting Information

Table S1 Relative influence of potential confounders on the relationship between parental misperception, and 1) parental intention to improve child engagement in overweight-related health behaviors, and 2) child meeting guidelines regarding these behaviors.
(DOCX)

Table S2 Mixed model logistic regression analyses, presenting the association between parental misperception and 1) parental intention to improve child engagement in overweight-related health behaviors, and 2) child meeting guidelines regarding these behaviors.
(DOCX)
