# Supplementary material for: Correlates of Parental Misperception of Their Child’s Weight Status: The ‘Be Active, Eat Right’ Study
Source: PLoS One. 2014 Feb 14;9(2):e88931. doi: 10.1371/journal.pone.0088931 (PMC3925202; doi:10.1371/journal.pone.0088931)
Supplement: Table S1 — Relative influence of potential confounders on the relationship between parental misperception, and 1) parental intention to improve child engagement in overweight-related health behaviors, and 2) child meeting guidelines regarding these behaviors. (DOCX) [file pone.0088931.s001.docx]

**Table S1** Relative influence of potential confounders on the relationship between parental misperception, and 1) parental intention to improve child engagement in overweight-related health behaviors, and 2) child meeting guidelines regarding these behaviors.

| Parental intention | | | |
| --- | --- | --- | --- |
| Playing outside | Having daily breakfast | Drinking sweet beverages | Watching TV |
| Child BMI | Child BMI | Child BMI | Child BMI |
| Parental country of birth | Parental country of birth | Parental country of birth | Parental country of birth |
|  |  |  | Parental gender |
| Child meeting guidelines | | | |
| Playing outside ≥ 1 hour per day | Having daily breakfast | Drinking ≤ 2 sweet beverages per day | Watching ≤ 2 hours of TV per day |
| Child BMI | Child BMI |  | Child BMI |
|  | Parental country of birth |  | Parental country of birth |
|  | Parental educational level |  |  |
