# Supplementary material for: Correlates of Parental Misperception of Their Child’s Weight Status: The ‘Be Active, Eat Right’ Study
Source: PLoS One. 2014 Feb 14;9(2):e88931. doi: 10.1371/journal.pone.0088931 (PMC3925202; doi:10.1371/journal.pone.0088931)
Supplement: Table S2 — Mixed model logistic regression analyses, presenting the association between parental misperception and 1) parental intention to improve child engagement in overweight-related health behaviors, and 2) child meeting guidelines regarding these behaviors. (DOCX) [file pone.0088931.s002.docx]

**Table S2.** Mixed model logistic regression analyses, presenting the association between parental misperception and 1) parental intention to improve child engagement in overweight-related health behaviors, and 2) child meeting guidelines regarding these behaviors.

| **Parental misperception and parental intention (agreed vs. not agreed with intention)** | | Playing outside | Having daily breakfast | Drinking sweet beverages | Watching TV |
| --- | --- | --- | --- | --- | --- |
|  | Crude OR (95% CI) | 0.84 (0.58 to 1.21) | 1.15 (0.79 to 1.66) | 0.94 (0.65 to 1.37) | 1.04 (0.72 to 1.50) |
|  | Adjusted OR (95% CI) $\ddagger$ | 0.83 (0.55 to 1.26) | 1.01 (0.67 to 1.53) | 0.90 (0.59 to 1.36) | 0.98 (0.64 to 1.49) |
|  | Adjusted OR (95% CI) $\dagger$ | 0.81 (0.52 to 1.24) | 0.94 (0.61 to 1.46) | 0.82 (0.54 to 1.27) | 0.92 (0.59 to 1.43) |
| **Parental misperception and child meeting guidelines (meeting guideline vs. not meeting guideline)** | | Playing outside ≥ 1 hour per day | Having daily breakfast | Drinking ≤ 2 sweet beverages per day | Watching ≤ 2 hours of TV per day |
|  | Crude OR (95% CI) | 1.16 (0.64 to 2.09) | 0.60 (0.38 to 0.93)* | 0.99 (0.73 to 1.35) | 0.64 (0.46 to 0.90)* |
|  | Adjusted OR (95% CI) $\ddagger$ | 1.29 (0.69 to 2.36) | 0.60 (0.36 to 0.98)* | 0.99 (0.73 to 1.35) | 0.67 (0.46 to 0.97)* |
|  | Adjusted OR (95% CI) $\dagger$ | 1.21 (0.63 to 2.31) | 0.64 (0.38 to 1.06) | 0.93 (0.65 to 1.32) | 0.85 (0.57 to 1.27) |

Note: YHC-cluster was entered as a random factor, with parental misperception and covariates were entered as fixed effects. $\ddagger$ adjusted for selected variables according to ‘change in estimate’ strategy, $\dagger$ adjusted for child age, gender, BMI and parental age, gender, country of birth, educational level, and weight status*, ** significant difference at *p*< 0.05.
